# Targeted Protein Degradation by Chimeric Compounds using Hydrophobic E3 Ligands and Adamantane Moiety

**DOI:** 10.3390/ph13030034

**Published:** 2020-02-25

**Authors:** Takuji Shoda, Nobumichi Ohoka, Genichiro Tsuji, Takuma Fujisato, Hideshi Inoue, Yosuke Demizu, Mikihiko Naito, Masaaki Kurihara

**Affiliations:** 1 Division of Organic Chemistry, National Institute of Health Sciences, 3-25-26 Tonomachi, Kawasaki, Kanagawa 210-9501, Japan; gtsuji@nihs.go.jp (G.T.); s116191@toyaku.ac.jp (T.F.); demizu@nihs.go.jp (Y.D.); mkurihara@iuhw.ac.jp (M.K.); 2 Division of Molecular Target and Gene Therapy Products, National Institute of Health Sciences, 3-25-26 Tonomachi, Kawasaki, Kanagawa 210-9501, Japan; n-ohoka@nihs.go.jp (N.O.); miki-naito@nihs.go.jp (M.N.); 3 School of Life Sciences, Tokyo University of Pharmacy and Life Sciences, 1432-1 Horinouchi, Hachioji, Tokyo 192-0392, Japan; hinoue@toyaku.ac.jp

**Keywords:** protein degradation, chimeric compound, hydrophobic tagging, adamantane

## Abstract

Targeted protein degradation using small chimeric molecules, such as proteolysis-targeting chimeras (PROTACs) and specific and nongenetic inhibitors of apoptosis protein [IAP]-dependent protein erasers (SNIPERs), is a promising technology in drug discovery. We recently developed a novel class of chimeric compounds that recruit the aryl hydrocarbon receptor (AhR) E3 ligase complex and induce the AhR-dependent degradation of target proteins. However, these chimeras contain a hydrophobic AhR E3 ligand, and thus, degrade target proteins even in cells that do not express AhR. In this study, we synthesized new compounds in which the AhR ligands were replaced with a hydrophobic adamantane moiety to investigate the mechanisms of AhR-independent degradation. Our results showed that the compounds, **2**, **3**, and **16** induced significant degradation of some target proteins in cells that do not express AhR, similar to the chimeras containing AhR ligands. However, in cells expressing AhR, **2**, **3**, and **16** did not induce the degradation of other target proteins, in contrast with their response to chimeras containing AhR ligands. Overall, it was suggested that target proteins susceptible to the hydrophobic tagging system are degraded by chimeras containing hydrophobic AhR ligands even without AhR.

## 1. Introduction

A technology for selectively degrading a target protein is expected not only to elucidate the physiological function of proteins but also to develop a therapeutic drug for a disease caused by the aberrant protein. Previous studies reported a series of chimeric compounds that recruit E3 ubiquitin ligases to specifically degrade target proteins via the ubiquitin-proteasome system [1,2]. These chimeras, which are called specific and nongenetic inhibitors of apoptosis protein [IAP]-dependent protein erasers (SNIPERs) and proteolysis-targeting chimeras (PROTACs), contain two ligands connected with a linker: one ligand specific to an E3 ubiquitin ligase and another ligand specific to the target protein. These chimeric compounds are designed to cross-link the E3 ligase with the target protein within the cells, and thereby, induce ubiquitination and subsequent degradation by proteasomes. Several chimeric degraders have successfully been employed to degrade several proteins such as nuclear receptors [3,4,5,6,7], oncogenic kinases [8], epigenetic regulators [9,10], transcription factors [11], and others [12,13]. Some compounds can also degrade target proteins in vivo, suggesting the possibility of clinical applications [5,9,10,14,15]. In contrast to traditional inhibitor-based pharmaceuticals, such as reversible/irreversible inhibitor and antagonists, the chimeras require only the transient binding to any surface of the target to catalytically induce its ubiquitination. Thus, this technology has emerged as a novel therapeutic approach known as “undruggable” proteins.

**Figure 1:**
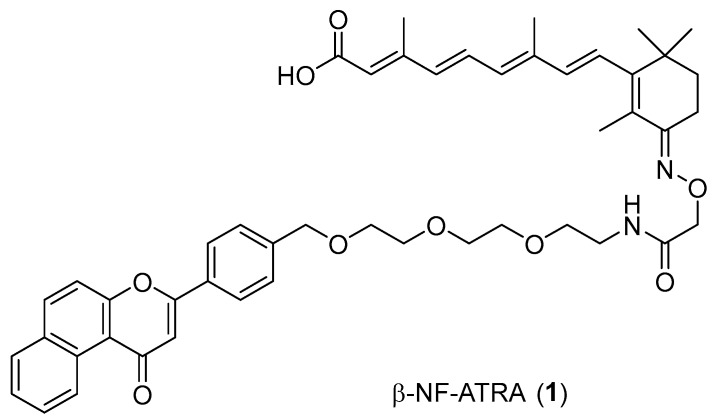
Chemical structure of β-NF-ATRA (**1**).

Recently, we developed a new class of small molecule chimeras that recruit the aryl hydrocarbon receptor (AhR) E3 ligase and induce the AhR-dependent degradation of target proteins [16]. β-NF-ATRA, **1** (Figure 1), a chimeric compound directed against cellular retinoic acid-binding proteins (CRABPs), induced the AhR-dependent degradation of CRABP-1 and CRABP-2 in MCF-7 and IMR-32 cells expressing AhR [16]. Interestingly, the **1**-induced significant reduction of CRABP-2, but not of CRABP-1, was also observed in SH-SY5Y cells, which do not express AhR (Figures S1 and S2). Similar results were also obtained by the treatment of the ITE-ATRA [16], in which an alternative AhR ligand was used (Figure S3). The ligand-bound AhR forms a CUL4B-based E3 ligase complex [17]. The NEDD8-activating E1 enzyme inhibitor MLN4924 [18,19] completely inhibited the **1**-induced reduction of CRABP-1 but partly that of CRABP-2 (Figure S4). These results suggested that these chimeric compounds degrade CRABP-1 and CRABP-2 via an AhR-dependent mechanism, whereas CRABP-2 is also degraded via an AhR-independent mechanism.

In this study, we synthesized several compounds to investigate the mechanisms of AhR-independent degradation and evaluate their activities. These results will provide useful information for the development of chimeric compounds using a highly hydrophobic E3 ligand and their pharmaceutical applications. 

## 2. Results and Discussion

### 2.1. Design and Synthesis of Compounds

**Figure 2:**
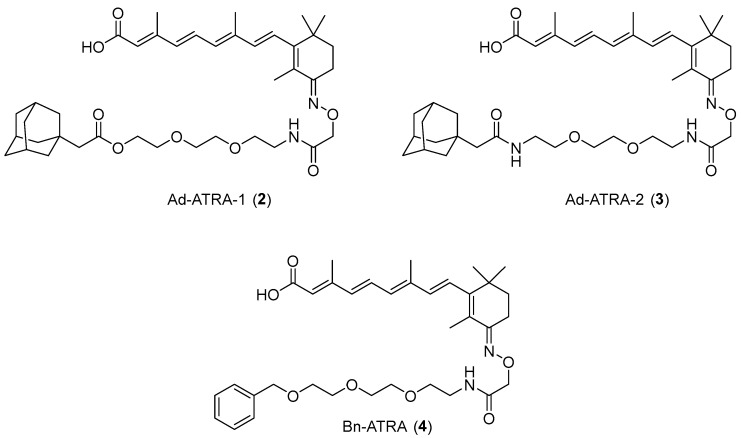
Chemical structures of designed compounds.

Since CRABP-2 was degraded by **1** and ITE-ATRA in SH-SY5Y cells that do not express AhR (Figures S1–S3), we assumed that the high hydrophobicity of the β-NF ligand module might play a role in the AhR-independent degradation of CRABP-2. Appending a hydrophobic moiety to a protein surface induces protein degradation; the hydrophobic moiety mimics a state of partial unfolding, inducing the degradation of the target protein via an intracellular quality control system [20,21,22]. Boc_3_-Arg- and adamantane-based hydrophobic tagging (HyT) strategies have been applied to a range of exogenous [23,24] and endogenous proteins [25,26]. To investigate whether CRABP proteins are degraded by the HyT system, we designed and synthesized **2** (Ad-ATRA-1) and **3** (Ad-ATRA-2) by conjugating an adamantane moiety (Figure 2). Besides, we designed **4** that lacked the benzochromene moiety of **1** because we expected that the hydrophobicity of **4** would be smaller than that of **1**.

**Scheme 1:**
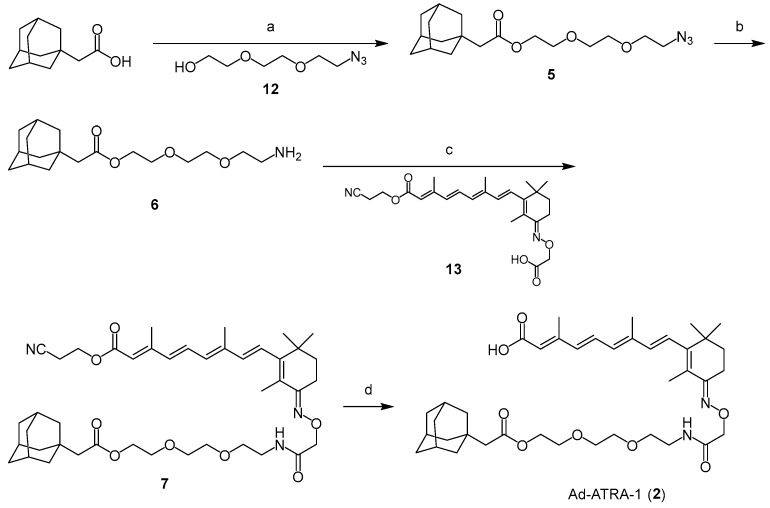
(**a**) 12, EDC-HCl, DMAP, CH_2_Cl_2_; (**b**) H_2_ gas, 10% Pd/C, EtOAc; (**c**) 13, EDC-HCl, DMAP, CH_2_Cl_2_; (**d**) TBAF, THF.

**Scheme 2:**
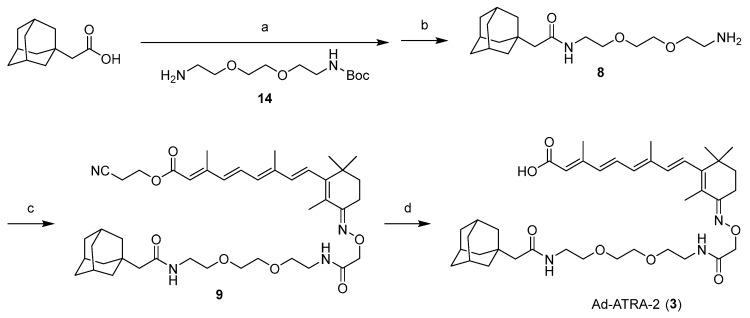
(**a**) 14, EDC-HCl, HOBt, CH_2_Cl_2_; (**b**) 4M HCl/1,4-dioxane; (**c**) 13, HATU, DIPEA, DMF; (**d**) TBAF, THF.

**Scheme 3:**
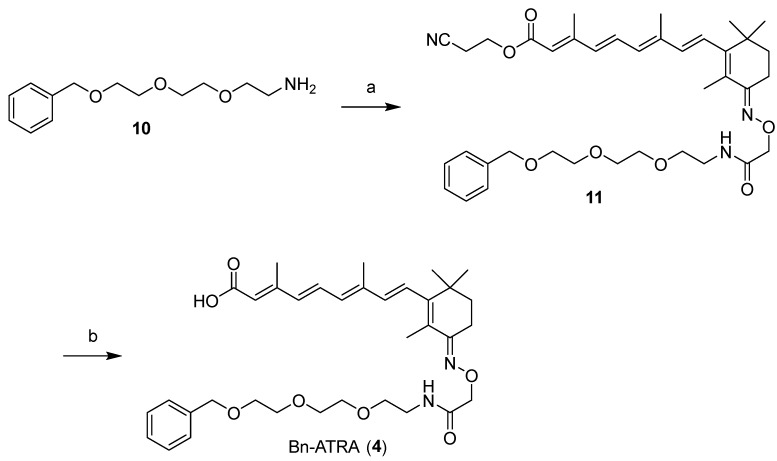
(**a**) 13, HATU, DIPEA, DMF; (**b**) TBAF, THF.

Scheme 1 presents the synthetic route of **2**. 1-Adamantanecarboxylic acid was condensed with **12** [27] to obtain **5**. Adamantane-ATRA, with an ester bond (Ad-ATRA-1, **2**), was obtained after the reduction of the azide group in **5**, the condensation with **13** [12], and the deprotection of **7**. Adamantane-ATRA, with an amide bond (Ad-ATRA-2, **3**) was also prepared via the condensation of **8** with **13**, following a similar procedure (Scheme 2). Bn-ATRA (**4**) was obtained by condensing the corresponding glycol amino linkers with **13**, followed by deprotection (Scheme 3).

### 2.2. Evaluation of Protein Degradation Activity

**Figure 3:**
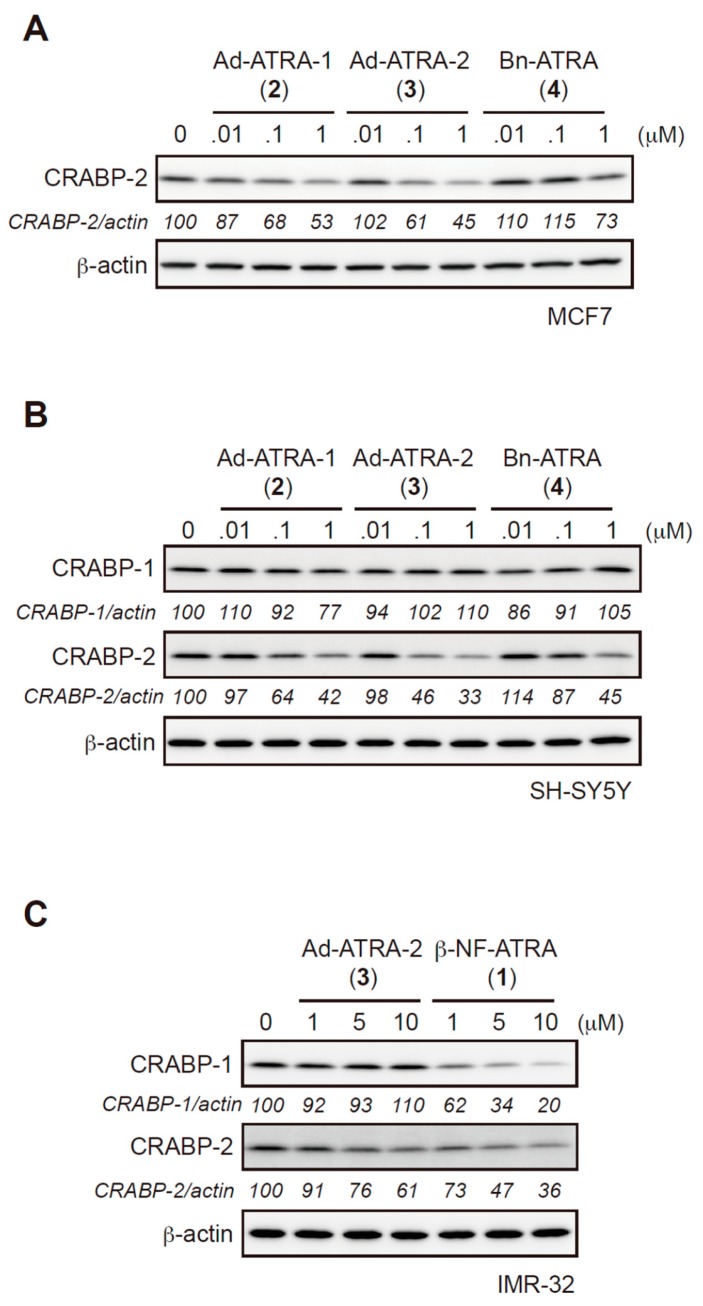
HyT compounds induce the degradation of CRABP-2 but not of CRABP-1. (**A**–**C**) Protein knockdown activities of HyT **2**, **3**, and **4** or β-NF-ATRA **1** in MCF-7 (**A**), SH-SY5Y (**B**), or IMR-32 cells (**C**). Cells were treated with the indicated compounds for 24 h. Whole-cell lysates were analyzed by western blotting. The numbers below the CRABP-1 and CRABP-2 panels represent CRABP-1/actin and CRABP-2/actin ratios, respectively, normalized by designating the expression from the vehicle control condition as 100%.

We examined the protein reduction activity of **2**, **3**, and **4** using various cell lines. In MCF-7 cells; **2** and **3** effectively reduced the CRABP-2 protein level, whereas **4**, which contained the less hydrophobic benzyl moiety, showed attenuated protein knockdown activity (Figure 3A and Figure S5). The CRABP2 reduction activity of **3** was similar to that of **1** in MCF7 cells (Figure S6). The CRABP2 knockdown activities of **2**, **3,** and **4** in SH-SY5Y cells were similar to their activities in MCF-7 cells (Figure 3B). The protein level of CRABP-1 was not significantly reduced by **2**, **3**, and **4** in SH-SY5Y cells (Figure 3B). Besides, **3** tended to reduce CRABP-2 in IMR-32 cells that expressed AhR but not CRABP-1 in contrast to **1** (Figure S1 and Figure 3C). These results indicated that CRABP-2, but not CRABP-1, was susceptible to HyT-mediated degradation. Thus, **1**, which contains the highly hydrophobic β-NF ligand, is likely to degrade CRABP-2 in the absence of AhR via the HyT mechanism.

**Figure 4:**
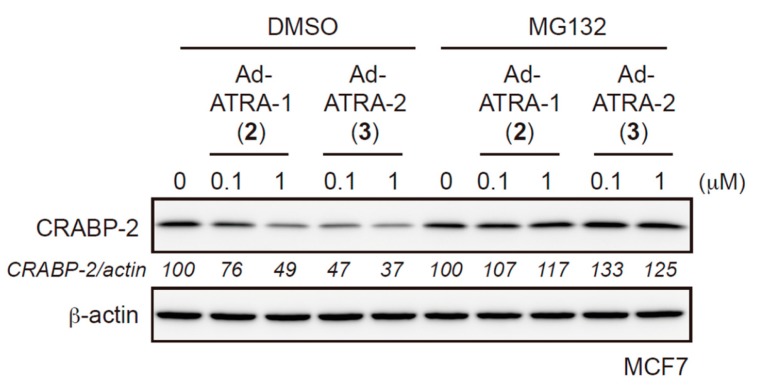
Proteasomal inhibitor MG132 inhibits the reduction of CRABP-2 by HyT compounds. MCF-7 cells were incubated with the indicated concentrations of HyT compounds in the presence or absence of 10 µM MG132 for 18 h. Whole-cell lysates were analyzed by western blotting.

We also examined the involvement of the proteasome in HyT-mediated degradation. The **2** and **3**-induced reductions of CRABP-2 were completely inhibited in the presence of the proteasome inhibitor MG132, suggesting that **2** and **3** induce the proteasomal degradation of CRABP-2 (Figure 4). This result clearly indicated the involvement of proteasome activity for the reduction of the target protein. However, this result could not deny the other possibilities, such as lysosomal degradations, or insolubilization by Lys63-linked ubiquitination. It is important to reveal the complexed pathway leading to the proteasome.

**Scheme 4:**
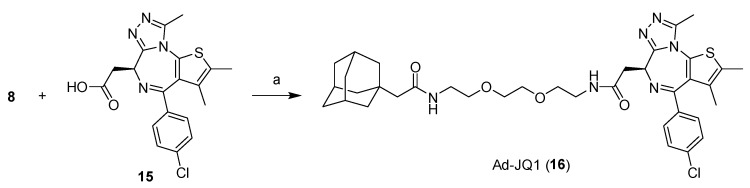
(a) HBTU, DIPEA, and DMF.

**Figure 5:**
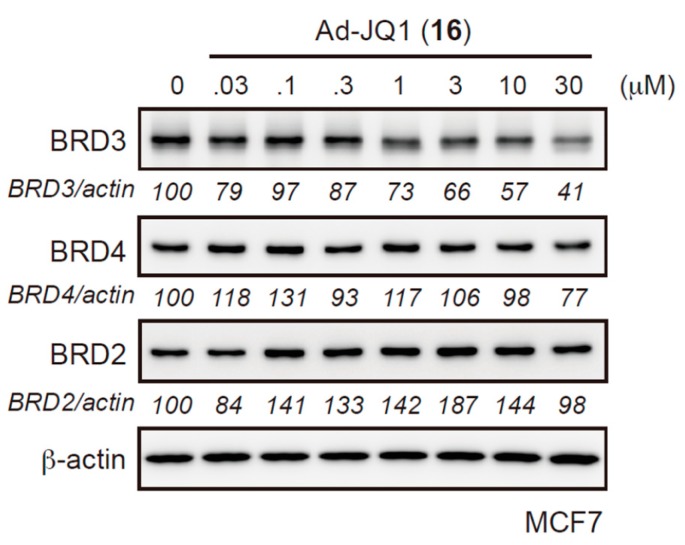
Ad-JQ1 (**16**) induces the degradation of BRD3 but not of BRD2 or BRD4. Protein knockdown activities of Ad-JQ1 in MCF-7 cells. Cells were treated with the indicated concentration of Ad-JQ1 for 48 h. Whole-cell lysates were analyzed by western blotting.

Next, we expanded the investigation to other target proteins. We previously developed the chimeric compound β-NF-JQ1 that is directed against bromodomain-containing (BRD) proteins using β-NF or (+)-JQ1 as an AhR or BRD ligand, respectively. β-NF-JQ1 induced the AhR-dependent degradation of BRD proteins in MCF-7 cells expressing AhR [16]. However, the β-NF-JQ1-induced reduction of BRD3, but not of BRD2 or BRD4, was also observed in SH-SY5Y cells, which do not express AhR (Figure S7), suggesting that BRD3 is also degraded by the AhR-independent mechanism. Therefore, we synthesized Ad-JQ1 (**15**) that conjugated an adamantane moiety to JQ1 (Scheme 4). Figure 5 presents the significant Ad-JQ1-induced reduction of BRD3, but not of BRD2 or BRD4 (Figure 5). These results suggested that BRD3, but not others, is susceptible to HyT-mediated degradation. 

In the present study, we designed and synthesized compounds with adamantane moiety to investigate whether the AhR-independent degradation of CRABP-2 and BRD3 by the chimeric compounds using AhR ligands are induced by the HyT system. We observed that **2, 3,** and **16** induced significant degradation of CRABP-2 and BRD3, but not of other target proteins, by the proteasome. Besides, Bn-ATRA **4,** which is less hydrophobic than **1**, **2**, and **3**, induced a weaker activity than **1**, **2**, and **3**. In conclusion, these results indicated that CRABP-2 and BRD3 are susceptible to HyT-mediated degradation and that the AhR-independent reduction of these proteins was caused by the HyT system. These results also provided useful information for drug development using chimeric compounds with a highly hydrophobic E3 ligand, which may occasionally induce targeted protein degradation by the HyT system. In addition to our findings, it is important to clarify the types of ubiquitin chains on target proteins and the specific E3 ligases involved in the ubiquitination.

## 3. Materials and Methods

All reagents and solvents were purchased from Sigma-Aldrich, Wako Pure Chemical, and Tokyo Chemical Industry and used without purification. Analytical TLC was conducted using Merck silica gel 60 F254 pre-coated plates, and visualized with a 254 nm UV lamp using phosphomolybdic acid, *p*-anisaldehyde, or ninhydrin stains. Column chromatography was performed using silica gel (spherical, neutral) purchased from Kanto Chemical. ^1^H and ^13^C NMR spectra were measured using a Varian AS 400 spectrometer or a JEOL ECZ 600R spectrometer, and measurements were carried out using deuterated solvents. Chemical shifts are expressed in ppm downfield from a solvent residual peak or the internal standard TMS. Mass spectra were measured using a Shimadzu IT-TOF MS equipped with an electrospray ionization source. 

**(2*E*,4*E*,6*E*,8*E*)-9-((*E*)-3-(((14-((3*r*,5*r*,7*r*)-adamantan-1-yl)-2,13-dioxo-6,9,12-trioxa-3-azatetradecyl)oxy)imino)-2,6,6-trimethylcyclohex-1-en-1-yl)-3,7-dimethylnona-2,4,6,8-tetraenoic acid** (Ad-ATRA-1, **2**)

EDC-HCl (120 mg, 0.63 mmol), followed by DMAP (15 mg, 0.12 mmol), was added to a mixture of 1-adamantaneacetic acid (100 mg, 0.52 mmol) and 2-(2-(2-azidoethoxy)ethoxy)ethan-1-ol (**12**) [27] (108 mg, 0.62 mmol) in CH_2_Cl_2_ (2 mL). After continuous stirring for two days at room temperature, the reaction mixture was diluted with EtOAc and washed with 2M HCl, saturated with aqueous NaHCO_3_ and brine. The organic layer was dried over Na_2_SO_4_ and concentrated under reduced pressure. The residue was purified by silica gel column chromatography (hexanes/EtOAc = 7:3) to obtain **5** as a colorless oil (47 mg, 26%). ^1^H NMR (400 MHz, CDCl_3_) δ 4.23–4.21 (m, 2H), 3.72–3.66 (m, 8H), 3.40–3.38 (m, 2H), 2.10 (s, 2H), 1.97 (br. s, 3H), 1.72–1.62 (m, 12H); ^13^C NMR (150 MHz, CDCl_3_) δ 171.8, 70.6, 70.5, 70.0, 69.2, 62.8, 50.5, 48.7, 42.2, 36.6, 32.7, 28.5. ESI-HRMS calcd for C_18_H_30_N_3_O_4_ [M+H]^+^: 352.2231, found: 352.2194.

A mixture of **5** (12 mg, 0.03 mmol) in EtOAc (10 mL) with 10% Pd/C (7.3 mg) was stirred for 13 h under H_2_ gas at room temperature and then, filtered through Celite to remove the catalyst. The filtrate was concentrated under reduced pressure to obtain the crude product (10.5 mg), which was used in the following step without further purification. EDC-HCl (14.6 mg, 0.076 mmol), followed by DMAP (9.3 mg, 0.076 mmol), was added to the mixture of the crude product (10.5 mg as 0.03 mmol) and **13** [12] (14.4 mg, 0.033 mmol) in CH_2_Cl_2_ (2 mL) After continuous stirring for 23 h at room temperature, the reaction mixture was diluted with CH_2_Cl_2_ and washed with brine. The organic layer was dried over Na_2_SO_4_ and concentrated under reduced pressure. The residue was purified by silica gel column chromatography (hexanes/EtOAc = 3:1) to obtain **7** as a yellow oil (7.9 mg, 31% for two steps). ^1^H NMR (400 MHz, CDCl_3_) δ 7.03 (dd, *J* = 15.0, 15.0 Hz, 1H), 6.67 (t, *J* = 5.4 Hz, 1H), 6.35 (d, *J* = 15.6 Hz, 1H), 6.33–6.30 (m, 1H), 6.22 (d, *J* = 15.0 Hz, 1H), 6.21 (d, *J* = 15.6 Hz, 1H), 5.82 (s, 1H), 4.59 (s, 2H), 4.33 (t, *J* = 6.0 Hz, 2H), 4.21 (t, *J* = 6.0 Hz, 2H), 3.67 (t, *J* = 4.8 Hz, 2H), 3.62–3.60 (m, 4H), 3.58 (t, *J* = 4.8 Hz, 2H), 3.54–3.51 (m, 2H), 2.74 (t, *J* = 6.0 Hz, 2H), 2.67 (t, *J* = 6.6 Hz, 2H), 2.37 (s, 3H), 2.09 (s, 2H), 2.03 (s, 3H), 1.96 (s, 3H), 1.87 (s, 3H), 1.73–1.69 (m, 2H), 1.63–1.61 (m, 12H), 1.10 (s, 6H); ^13^C NMR (150 MHz, CDCl_3_) δ 171.6, 170.1, 166.1, 158.6, 154.4, 150.0, 139.1, 139.1, 135.9, 131.3, 127.0, 125.0, 117.6, 117.0, 73.1, 70.4, 70.3, 70.0, 69.2, 62.8, 58.0, 48.8, 42.3, 38.6, 38.6, 36.7, 36.0, 34.8, 32.7, 28.6, 27.6, 20.2, 18.1, 14.8, 14.0, 12.8. ESI-HRMS calcd for C_43_H_62_N_3_O_8_ [M+H]^+^: 748.4531, found: 748.4529.

TBAF (1 M in THF solution, 43 µL, 0.043 mmol) was added to a solution of **7** (8 mg, 0.010 mmol) in THF (0.19 mL). After continuous stirring for 1.5 h at room temperature, the reaction mixture was diluted with CHCl_3_, containing 0.1% AcOH and then, concentrated under reduced pressure. The residue was purified by flash silica gel column chromatography (CHCl_3_/MeOH = 99:1 to 98:2) to obtain **2** as a yellow solid (4.0 mg, 54%, containing ca. 10% of the 13-*cis* isomer). ^1^H NMR (400 MHz, CDCl_3_) δ 7.03 (dd, *J* = 15.6, 15.6 Hz, 1H), 6.68 (t, *J* = 5.7 Hz, 1H), 6.36 (d, *J* = 15.0 Hz, 1H), 6.31 (d, *J* = 16.2 Hz, 1H), 6.23–6.21 (m, 2H), 5.83 (s, 1H), 4.59 (s, 2H), 4.21 (t, *J* = 5.1 Hz, 2H), 3.67 (t, *J* = 4.8 Hz, 2H), 3.61–3.52 (m, 8H), 2.67 (t, *J* = 6.6 Hz, 2H), 2.37 (s, 3H), 2.03 (s, 3H), 1.96 (br., 4H), 1.87 (s, 3H), 1.69 (d, *J* = 12.0 Hz, 3H), 1.63–1.61 (m, 12H), 1.10 (s, 6H); ^13^C NMR (150 MHz, CDCl_3_) δ 171.7, 170.6, 170.2, 158.7, 154.7, 150.1, 139.2, 139.0, 136.2, 131.4, 131.2, 127.0, 125.0, 118.1, 73.1, 70.4, 70.3, 69.9, 69.3, 62.8, 48.8, 42.3, 38.7, 36.7, 36.0, 34.9, 32.8, 28.6, 27.6, 20.2, 14.9, 14.0, 12.9. ESI-HRMS calcd for C_40_H_59_N_2_O_8_ [M+H]^+^: 695.4266, found: 695.4226.

**(2*E*,4*E*,6*E*,8*E*)-9-((*E*)-3-(((14-((3*r*,5*r*,7*r*)-adamantan-1-yl)-2,13-dioxo-6,9-dioxa-3,12-diazatetradecyl)oxy)imino)-2,6,6-trimethylcyclohex-1-en-1-yl)-3,7-dimethylnona-2,4,6,8-tetraenoic acid** (Ad-ATRA-2, **3**)

EDC-HCl (144 mg, 0.75 mmol) was added to a mixture of 1-adamantaneacetic acid (97 mg, 0.50 mmol), *N*-(*tert*-butoxycarbonyl)-2,2′-(ethylenedioxy)diethylamine **14** (149 mg, 0.60 mmol) and HOBt-H_2_O (0.75 mmol) in CH_2_Cl_2_ (1.0 mL). After continuous stirring for 1.5 h at room temperature, the reaction mixture was diluted with EtOAc and washed with 1M HCl, saturated aqueous NaHCO_3_ and brine. The organic layer was dried over Na_2_SO_4_ and concentrated under reduced pressure to give a colorless oil (238 mg). This crude product (200 mg, as 0.47 mmol) was treated with 4M HCl/1,4-dioxane (1 mL) for 8 h at room temperature. The volatiles were removed by evaporation under reduced pressure and subsequent co-evaporation with CH_3_CN (1 mL × 2) under reduced pressure. HCl salt was obtained as a colorless oil (**8**), which was used in the following reaction without further purification. HATU (76 mg, 0.20 mmol), followed by DIPEA (104 µL, 0.60 mmol), was added to a mixture of **13** (44 mg, 0.10 mmol) and **8** (54 mg, as 0.15 mmol) in DMF (0.3 mL). After continuous stirring for 12 h at room temperature, the reaction mixture was diluted with EtOAc and washed with 1 M HCl, saturated with aqueous NaHCO_3_ and brine. The organic layer was dried over Na_2_SO_4_ and concentrated under reduced pressure. The residue was purified by silica gel column chromatography (CHCl_3_/MeOH = 100:0 to 99:1) to obtain **9** as a yellowish amorphous solid (56 mg, 75%). ^1^H NMR (400 MHz, CDCl_3_) δ 7.04 (dd, *J* = 15.6, 15.0 Hz, 1H), 6.67 (t, *J* = 5.4 Hz, 1H), 6.35 (d, *J* = 15.0 Hz, 1H), 6.32 (t, *J* = 15.6 Hz, 1H), 6.24–6.21 (m, 2H), 5.91 (br. s, 1H), 5.83 (s, 1H), 4.59 (s, 2H), 4.33 (t, *J* = 6.0 Hz, 2H), 3.62–3.52 (m, 10H), 3.47–3.42 (m, 2H), 2.74 (t, *J* = 6.0 Hz, 2H), 2.67 (t, *J* = 6.6 Hz, 1H), 2.37 (s, 3H), 2.04 (s, 3H), 1.96 (s, 3H), 1.93 (s, 2H), 1.87 (s, 3H), 1.71–1.69 (m, 3H), 1.63–1.62 (m, 12H), 1.11 (s, 6H); ^13^C NMR (150 MHz, CDCl_3_) δ 170.9, 170.2, 166.1, 158.7, 154.3, 150.1, 139.1, 139.0, 135.9, 131.3, 131.2, 126.9, 124.8, 117.5, 116.9, 73.0, 70.2, 70.1, 70.0, 69.8, 58.0, 51.6, 42.5, 38.9, 38.6, 36.7, 35.9, 34.8, 32.6, 28.6, 27.5, 20.1, 18.0, 14.8, 13.9, 12.8. ESI-HRMS calcd for C_43_H_63_N_4_O_7_ [M+H]^+^: 747.4691, found: 747.4655.

TBAF (1 M in THF solution, 117 µL, 117.0 µmol) was added to a solution of **9** (29 mg, 0.039 mmol) in THF (0.7 mL). After continuous stirring for 1.5 h at room temperature, the reaction mixture was concentrated under reduced pressure. The residue was purified by flash silica gel column chromatography (CHCl_3_/MeOH = 100:0 to 98:2) to obtain **3** as a yellowish amorphous solid (11 mg, 42%, containing 10% of the 13-*cis* isomer). ^1^H NMR (400 MHz, CDCl_3_) δ 7.02 (dd, *J* = 15.0, 15.0 Hz, 1H), 6.69 (t, *J* = 6.0 Hz, 1H), 6.34 (d, *J* = 15.0 Hz, 1H), 6.32 (d, *J* = 16.8 Hz, 1H), 6.29–6.19 (m, 2H), 5.93 (br. t, 1H), 5.83 (s, 1H), 4.60 (s, 2H), 3.62–3.53 (m, 10H), 3.45 (t, *J* = 5.6 Hz, 2H), 2.67 (t, *J* = 6.6 Hz, 2H), 2.37 (s, 3H), 2.03 (s, 3H), 1.96 (br. s, 3H), 1.94 (s, 2H), 1.87 (s, 3H), 1.69 (d, *J* = 12.0 Hz, 3H), 1.63–1.61 (m, 11H), 1.10 (s, 6H); ^13^C NMR (150 MHz, CDCl_3_) δ 171.1, 170.8, 170.4, 158.8, 154.3, 150.2, 139.2, 138.9, 136.3, 131.5, 131.1, 126.8, 124.8, 118.5, 73.0, 70.2, 70.1 (1C overlapped), 69.9, 51.7, 42.6, 39.0, 38.7, 36.7, 35.9, 34.8, 32.7, 28.6, 27.6, 20.2, 14.9, 14.0, 12.8. ESI-HRMS calcd for C_40_H_60_N_3_O_7_ [M+H]^+^: 694.4426, found: 694.4391. 

**(2*E*,4*E*,6*E*,8*E*)-3,7-dimethyl-9-((*E*)-2,6,6-trimethyl-3-(((12-oxo-1-phenyl-2,5,8-trioxa-11-azatridecan-13-yl)oxy)imino)cyclohex-1-en-1-yl)nona-2,4,6,8-tetraenoic acid)** (Bn-ATRA, **4**)

HATU (46 mg, 0.12 mmol), followed by DIPEA (42 µL, 0.24 mmol), were added to a mixture of **13** (44 mg, 0.10 mmol) and **10** [28,29] (48 mg, 0.20 mmol) in DMF (2 mL). After continuous stirring for 12 h at room temperature, the reaction mixture was diluted with EtOAc and washed with 1 M HCl, saturated with aqueous NaHCO_3_ and brine. The organic layer was dried over Na_2_SO_4_ and concentrated under reduced pressure. The residue was purified by silica gel column chromatography (CHCl_3_/MeOH = 100:0 to 99:1) to obtain **11** as a yellow oil (41 mg, 62%). ^1^H NMR (600 MHz, CDCl_3_) δ 7.34–7.33 (m, 4H), 7.29–7.27 (m, 1H), 7.03 (dd, *J* = 15.6, 15.0 Hz, 1H), 6.69 (br. t, *J* = 5.4 Hz, 1H), 6.33 (d, *J* = 15.0 Hz, 1H), 6.31 (d, *J* = 15.6 Hz, 1H), 6.22 (d, *J* = 15.0 Hz, 1H), 6.22 (d, *J* = 15.0 Hz, 1H), 5.82 (s, 1H), 4.58 (s, 2H), 4.56 (s, 2H), 4.32 (t, *J* = 6.0 Hz, 2H), 3.67–3.65 (m, 2H), 3.64–3.62 (m, 6H), 3.59–3.57 (m, 2H), 3.53–3.51 (m, 2H), 2.74 (t, *J* = 6.0 Hz, 2H), 2.66 (t, *J* = 6.6 Hz, 2H), 2.37 (s, 3H), 2.03 (s, 3H), 1.87 (s, 3H), 1.61 (t, *J* = 6.6 Hz, 2H), 1.09 (s, 6H); ^13^C NMR (150 MHz, CDCl_3_) δ 170.0, 166.1, 158.5, 154.3, 149.9, 139.0, 138.0, 135.8, 131.3, 131.2, 128.3, 127.6, 127.5, 127.0, 124.9, 117.5, 117.0, 73.2, 73.0, 70.6, 70.4, 70.2, 69.8, 69.3, 58.0, 38.6, 38.5, 35.9, 34.8, 27.5, 20.1, 18.0, 14.8, 13.9, 12.8. ESI-HRMS calcd for C_38_H_52_N_3_O_7_ [M+H]^+^: 662.3800, found: 662.3834. 

TBAF (1 M in THF solution, 99 µL, 0.099 mmol) was added to a solution of **11** (22 mg, 0.033 mmol) in THF (0.6 mL). After continuous stirring for 1.5 h at room temperature, the reaction mixture was diluted with CHCl_3_, containing 0.1% AcOH, and then, concentrated under reduced pressure. The residue was purified by flash silica gel column chromatography (CHCl_3_/MeOH = 100:0 to 98:2) to obtain **4** as a yellow solid (8.6 mg, 44%, containing ca. 10% of the 13-*cis* isomer). ^1^H NMR (400 MHz, CDCl_3_) δ 7.34–7.33 (m, 5H), 7.06–6.99 (m, 1H), 6.66 (br. t, *J* = 5.6 Hz, 1H), 6.37–6.19 (m, 4H), 5.83 (s, 1H), 4.58 (s, 2H), 4.56 (s, 2H), 3.66–3.62 (m, 8H), 3.59–3.57 (m, 2H), 3.54–3.49 (m, 2H), 2.66 (t, *J* = 6.8 Hz, 2H), 2.37 (s, 3H), 2.02 (s, 3H), 1.87 (s, 3H), 1.61 (t, *J* = 6.8 Hz, 2H), 1.10 (s, 6H); ^13^C NMR (150 MHz, CDCl_3_) δ 171.3, 170.3, 158.7, 154.6, 150.0, 139.1, 139.0, 138.1, 136.2, 131.4, 131.2, 128.4, 127.7, 127.6, 127.0, 125.0, 118.4, 73.3, 73.1, 70.6, 70.5, 70.3, 69.9, 69.4, 38.7, 36.0, 34.8, 27.6, 20.2, 14.9, 14.0, 12.8. ESI-HRMS calcd for C_35_H_49_N_2_O_7_ [M+H]^+^: 609.3534, found: 609.3497.

**2-((3*r*,5*r*,7*r*)-adamantan-1-yl)-*N*-(2-(2-(2-(2-((*S*)-4-(4-chlorophenyl)-2,3,9-trimethyl-6*H*-thieno[3,2-*f*][1,2,4]triazolo[4,3-*a*][1,4]diazepin-6-yl)acetamido)ethoxy)ethoxy)ethyl)acetamide** (Ad-JQ1, **16**)

HBTU (21 mg, 0.055 mmol), followed by DIPEA (19.6 mg, 0.15 mmol), was added to a mixture of **8** (10.8 mg, 0.03 mmol) and **15** [10] in DMF (2 mL). After continuous stirring for 0.5 h, the reaction mixture was diluted with EtOAc and washed with 1M HCl, saturated aqueous NaHCO_3_, and brine. The organic layer was dried over Na_2_SO_4_ and concentrated under reduced pressure. The residue was purified by silica gel column chromatography (CHCl_3_/MeOH = 100:0 to 99:1) to obtain **16** as a yellow oil (6.0 mg, 34%). ^1^H NMR (600 MHz, CDCl_3_) δ 7.40 (d, *J* = 8.5 Hz, 2H), 7.32 (d, *J* = 8.9 Hz, 2H), 7.09 (dd, *J* = 5.4 Hz, 1H), 6.52–6.46 (m, 1H), 4.63 (dd, *J* = 8.1, 6.1 Hz, 1H), 3.66–3.53 (m, 10H), 3.52–3.41 (m, 3H), 3.32 (dd, *J* = 14.3, 6.1 Hz, 1H), 2.65 (s, 3H), 2.39 (s, 3H), 1.90 (s, 2H), 1.89–1.86 (m, 3H), 1.67 (d, *J* = 0.9 Hz, 3H), 1.65–1.53 (m, 12H). ^13^C NMR (150 MHz, CDCl_3_) δ 171.41, 170.57, 164.03, 155.74, 149.93, 136.94, 136.64, 132.15, 131.03, 131.00, 130.62, 129.93, 128.81, 77.32, 77.10, 76.89, 70.40, 70.37, 69.89, 54.61, 51.55, 42.71, 42.61, 39.47, 39.18, 36.87, 32.75, 28.73, 14.48, 13.19, 11.90, 0.08. ESI-HRMS calcd for C_37_H_48_N_6_ClO_4_S [M+H]^+^: 707.3141, found: 707.3210.

### 3.1. Cell Culture

Human breast carcinoma MCF-7 cells were maintained in RPMI 1640 medium containing 10% fetal bovine serum (FBS) and 100 µg ml^-1^ kanamycin. Human neuroblastoma SH-SY5Y cells were maintained in Dulbecco’s modified Eagle’s medium, containing 10% FBS and 100 µg ml^-1^ kanamycin. Human neuroblastoma IMR-32 cells were maintained in Eagle’s minimum essential medium, containing 10% FBS and 100 µg ml^-1^ kanamycin. Cells were treated with various concentrations of compounds for the indicated duration.

### 3.2. Western Blotting

Cells were lysed with SDS lysis buffer (0.1 M Tris-HCl at pH 8.0, 10% glycerol, 1% SDS) and immediately boiled for 10 min to obtain clear lysates. Protein concentrations were measured using the BCA method (Pierce). Lysates containing equal amounts of proteins were separated by SDS-PAGE and transferred to PVDF membranes (Millipore, MA, USA) for western blot analysis using the appropriate antibodies. Immunoreactive proteins were visualized using the Immobilon Western chemiluminescent HRP substrate (Millipore, MA, USA) or the Clarity Western ECL substrate (Bio-Rad, CA, USA). Light emission intensity was quantified using a LAS-3000 luminous-image analyzer equipped with Image Gauge 2.3 (Fuji, Japan). The antibodies used in this study were: anti-CRABP-2 rabbit polyclonal antibody (pAb) (Bethyl, A300-809A), anti-CRABP-1 rabbit monoclonal antibody (mAb) (Cell Signaling Technology, 13206), and anti-AhR rabbit pAb (Cell Signaling Technology, 13790). All Western blotting were performed several times to confirm reproducibility. The typical results were shown here.

### 3.3. Cell Viability Assay

Cell viability was determined using water-soluble tetrazolium WST-8 (2-(2-methoxy-4-nitrophenyl)-3-(4-nitrophenyl)-5-(2,4-disulfophenyl)-2*H*-tetrazolium) in a spectrophotometric assay, according to the manufacturer’s instructions (Dojindo, Japan). Cells treated with compounds were incubated with WST-8 for 0.5 h at 37 °C in a humidified atmosphere of 5% CO_2_. The absorbance at 450 nm was measured using an EnVision Multilabel Plate Reader (PerkinElmer, USA).

## Supplementary Materials

The following is available online at https://www.mdpi.com/1424-8247/13/3/34/s1, Figure S1: Levels of CRABP-1, CRABP-2, and AhR in the indicated cancer cells, Figure S2: Protein knockdown activity of β-NF-ATRA. SH-SY5Y cells were treated with β-NF-ATRA for 24 h. Whole-cell lysates were analyzed by western blotting, Figure S3: Chemical structure and protein knockdown activity of ITE-ATRA. SH-SY5Y cells were treated with ITE-ATRA for 8 h. Whole-cell lysates were analyzed by western blotting, Figure S4: Inhibitory effect of MLN4924 on IMR-32 cells. Cells were treated with β-NF-ATRA in the presence or absence of 10 µM MLN4924, Figure S5: Statistical analysis of protein knockdown activityof HyT 2, 3, and 4.in MCF-7 cells, Figure S6: Protein knockdown activity of the indicated compounds. MCF-7 cells were treated with the compounds for 24 h. Whole-cell lysates were analyzed by western blotting, Figure S7: Chemical structure and protein knockdown activity of β-NF-JQ1. SH-SY5Y cells were treated with β-NF-JQ1 for 24 h. Whole-cell lysates were analyzed by western blotting.

## Abbreviations

PROTAC	proteolysis-targeting chimeras
SNIPER	specific and nongenetic inhibitors of apoptosis protein [IAP]-dependent protein erasers
AhR	aryl hydrocarbon receptor
β-NF	β-naphthoflavone
ATRA	all-trans retinoic acid
CRABPs	cellular retinoic acid-binding proteins
ITE	2-(1′*H*-indole-3′-carbonyl)-thiazole-4-carboxylic acid methyl ester
HyT	hydrophobic tagging
Ad	adamantane
HATU	1-[bis(dimethylamino)methylene]-1*H*-1,2,3-triazolo[4,5-*b*]pyridinium 3-Oxide Hexafluorophosphate
DIPEA	*N*,*N*-diisopropylethylamine
DMF	*N*,*N*-dimethylformamide
TBAF	tetrabutylammonium Fluoride
THF	tetrahydrofuran
HBTU	1-[bis(dimethylamino)methylene]-1*H*-benzotriazolium 3-Oxide Hexafluorophosphate
TLC	thin-layer chromatography
UV	ultra-violet
NMR	nuclear magnetic resonance
TMS	tetramethylsilane
IT-TOF MS	ion trap-time-of-flight mass spectrometry
calcd	caluculated
DMAP	N,N-dimethyl-4-aminopyridine
