# Supplementary material for: Targeted Protein Degradation by Chimeric Compounds using Hydrophobic E3 Ligands and Adamantane Moiety
Source: Pharmaceuticals (Basel). 2020 Feb 25;13(3):34. doi: 10.3390/ph13030034 (PMC7151680; doi:10.3390/ph13030034)
Supplement: Supplementary file 1 [file pharmaceuticals-13-00034-s001.pdf]

Supplementary Material

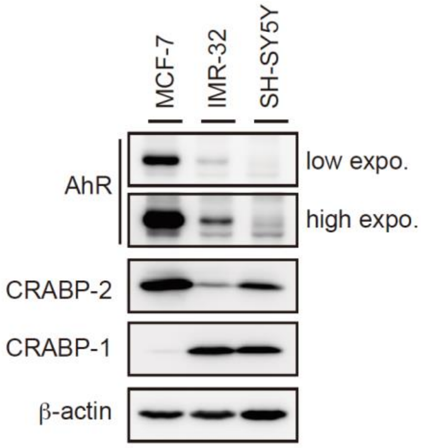

Figure S1

Levels of CRABP-1, CRABP-2, and AhR in the indicated cancer cells.

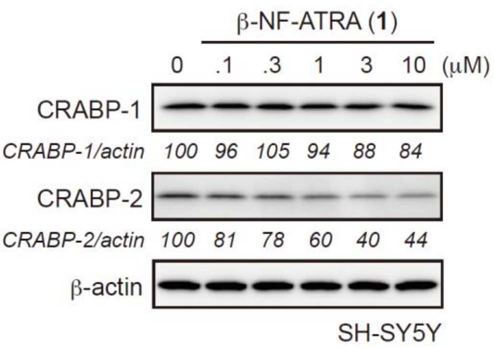

Figure S2

Protein knockdown activity of β-NF-ATRA. SH-SY5Y cells were treated with β-NF-ATRA for 24 h. Whole-cell lysates were analyzed by western blotting.

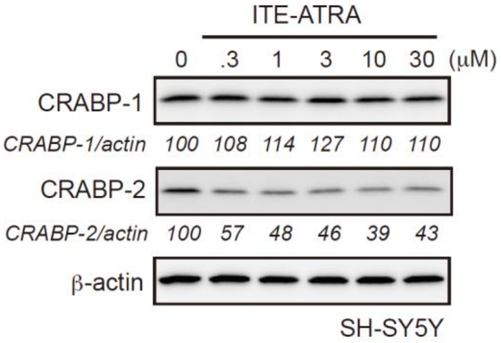

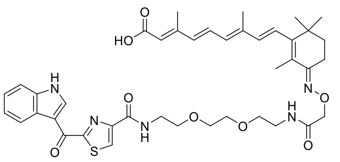

ITE-ATRA

Figure S3

Chemical structure and protein knockdown activity of ITE-ATRA. SH-SY5Y cells were treated with ITE-ATRA for 8 h. Whole-cell lysates were analyzed by western blotting.

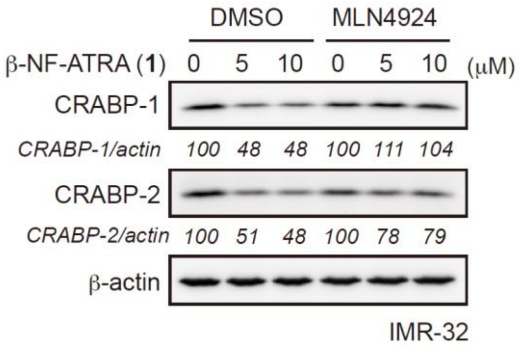

Figure S4
Inhibitory effect of MLN4924 on IMR-32 cells. Cells were treated with β-NF-ATRA in the presence or absence of 10 μM MLN4924.

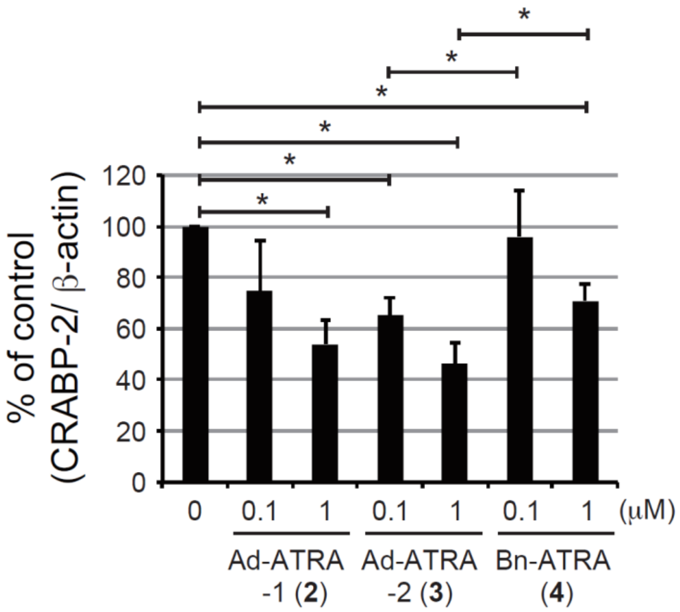

Figure S5
Statistical analysis of protein knockdown activityof HyT 2, 3, and 4.in MCF-7 cells.
Data in the bar graph are the mean ± S.D. (error bars) of three independent experiments.
* indicates $P < 0.05$

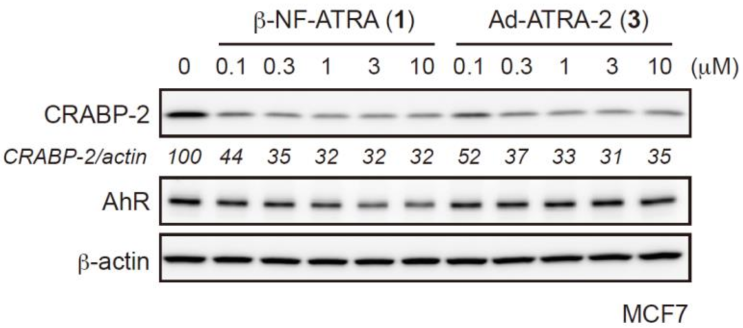

Figure S6

Protein knockdown activity of the indicated compounds. MCF-7 cells were treated with the compounds for 24 h. Whole-cell lysates were analyzed by western blotting.

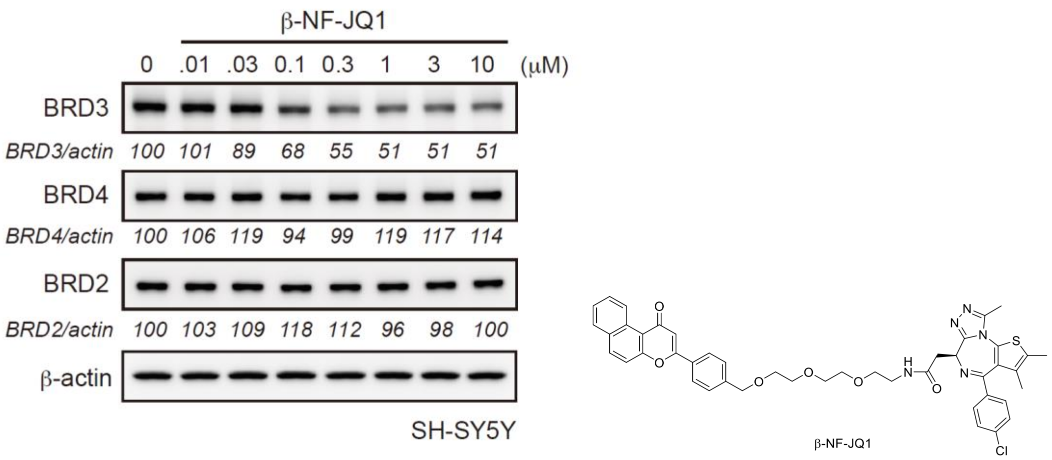

Figure S7
Chemical structure and protein knockdown activity of β-NF-JQ1. SH-SY5Y cells were treated with β-NF-JQ1 for 24 h. Whole-cell lysates were analyzed by western blotting.
